# Author Correction: Mutually suppressive roles of KMT2A and KDM5C in behaviour, neuronal structure, and histone H3K4 methylation

**DOI:** 10.1038/s42003-020-1061-7

**Published:** 2020-06-22

**Authors:** Christina N. Vallianatos, Brynne Raines, Robert S. Porter, Katherine M. Bonefas, Michael C. Wu, Patricia M. Garay, Katie M. Collette, Young Ah Seo, Yali Dou, Catherine E. Keegan, Natalie C. Tronson, Shigeki Iwase

**Affiliations:** 1 Department of Human Genetics, Michigan Medicine, University of Michigan, Ann Arbor, MI 48109 USA; 2 Genetics and Genomics Graduate Program, University of Michigan, Ann Arbor, MI 48109 USA; 3 Department of Psychology, University of Michigan, Ann Arbor, MI 48109 USA; 4 The University of Michigan Neuroscience Graduate Program, Ann Arbor, MI USA; 5 Neurodigitech, LLC, San Diego, CA 92126 USA; 6 Department of Nutritional Sciences, School of Public Health, University of Michigan, Ann Arbor, MI 48109 USA; 7 Department of Pathology, Michigan Medicine, University of Michigan, Ann Arbor, MI 48109 USA; 8 Department of Pediatrics, Michigan Medicine, University of Michigan, Ann Arbor, MI 48109 USA

**Keywords:** Autism spectrum disorders, Molecular neuroscience

Correction to: *Communications Biology* 10.1038/s42003-020-1001-6, published online 1 June 2020.

In the original published version of the Supplementary Information file, Supplementary Figure 10 was inadvertently omitted, although the figure caption was present. This has been corrected in the Supplementary Information file online.

