# Supplementary material for: Mutually suppressive roles of KMT2A and KDM5C in behaviour, neuronal structure, and histone H3K4 methylation
Source: Commun Biol. 2020 Jun 1;3:278. doi: 10.1038/s42003-020-1001-6 (PMC7264178; doi:10.1038/s42003-020-1001-6)
Supplement: Supplementary file 1 — Description of Additional Supplementary Files [file 42003_2020_1001_MOESM1_ESM.pdf]

Description of Additional Supplementary Files

Supplementary Data 1: List of differentially-expressed genes (DEGs) identified in the study.

Supplementary Data 2: List of rescue-driving genes identified in the RNA-seq study.

Supplementary Data 3: Ages of mice used in the study.

Supplementary Data 4: Source data of body weight represented in Fig. 1d.

Supplementary Data 5: Source data of behavioral studies presented in Fig. 2 and Fig. 3.

Supplementary Data 6: Source data of dendritic morphology analysis presented in Fig. 4.
