# Supplementary material for: Mutually suppressive roles of KMT2A and KDM5C in behaviour, neuronal structure, and histone H3K4 methylation
Source: Commun Biol. 2020 Jun 1;3:278. doi: 10.1038/s42003-020-1001-6 (PMC7264178; doi:10.1038/s42003-020-1001-6)
Supplement: Supplementary file 9 — Supplementary Information [file 42003_2020_1001_MOESM9_ESM.pdf]

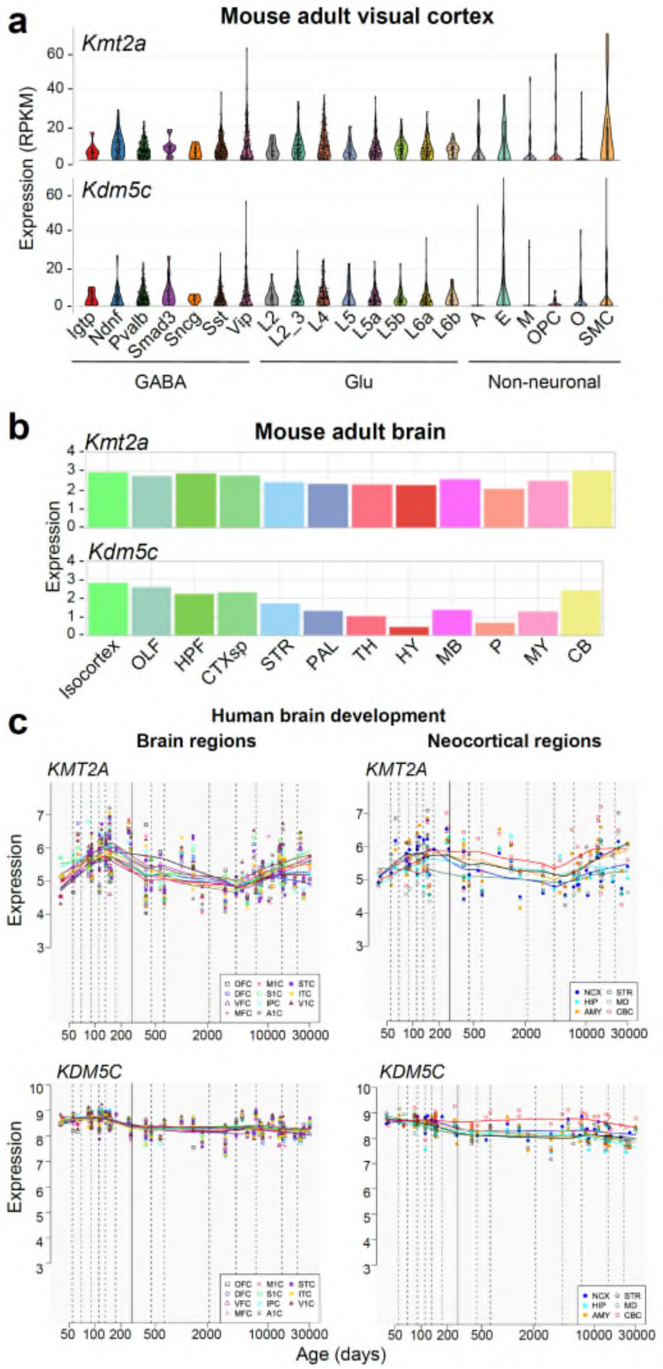

Supplementary Figure 1. Expression of KMT2A and KDM5C. (A) Expression of *Kmt2a* and *Kdm5c*, from FACS-sorted single cells of mouse visual cortex, shown in reads per kilobase of transcript per million mapped reads (RPKM). Neuronal cells: GABAergic (GABA), Glutamatergic (Glu). Non-neuronal cells: astrocytes (A); endothelial cells (E); microglia (M), oligodendrocyte precursor cells (OPC); oligodendrocytes (O); smooth muscle cells (SMC). Image credit: Broad Institute "Single Cell Portal" transcriptome of adult mouse visual cortex (1). **(B)** Expression of *Kmt2a* and *Kdm5c* mRNA from adult mouse brain, shown in log2 of raw expression value from *in situ* hybridization. Brain regions: Isocortex, olfactory areas (OLF), hippocampal formation (HPF), cortical subplate (CTXsp), striatum (STR), pallidum (PAL), thalamus (TH), hypothalamus (HY), midbrain (MB), pons (P), medulla (MY), cerebellum (CB). Image credit: Allen Institute, Allen Mouse Brain Atlas (2004) (2). **(C)** Expression of *KMT2A* and *KDM5C* transcripts, from developing and adult human brains, shown in RPKM. Human development and adulthood were split into the following Periods: 1-7 fetal development; 8-9 birth and infancy; 10-11 childhood; 12 adolescence; and 13-15 adulthood. Image credit: Human Brain Transcriptome Atlas (3, 4)

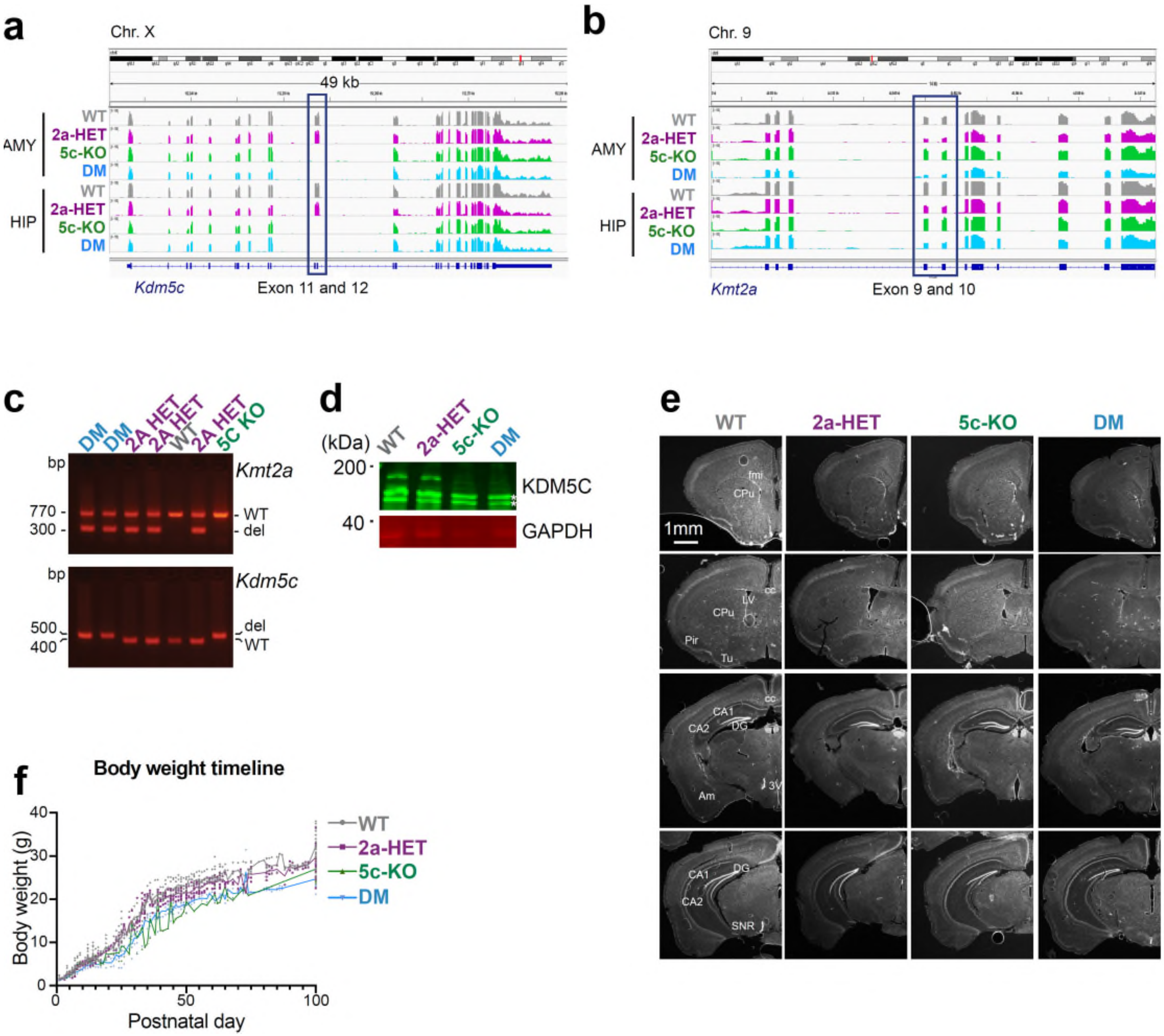

Supplementary Figure 2. Basic features of mutant mice. (A-B) RNA-seq read coverage of *Kmt2a* **(A)** and *Kdm5c* **(B)** genes and targeted exons (highlight) confirmed the intended gene manipulations. **(C)** Genotyping using genomic DNA, confirming presence of *Kmt2a* and/or *Kdm5c* deleted alleles ("del") only in appropriate genotypes. **(D)** Western blot for KDM5C protein. Stars indicate non-specific bands present in all samples. GAPDH shown for equal loading. **(E)** Serial brain sections 30 μm thick stained with DAPI to mark nuclei. Sections shown at Bregma regions 1.41, 0.49, -2.15, and -2.91 mm (top to bottom). Regions highlighted: anterior forceps of the corpus callosum (fmi), caudate putamen (CPu), corpus callosum (cc), lateral ventricle (LV), piriform cortex (Pir), olfactory tubercle (Tu), hippocampal fields CA1 and CA2, dentate gyrus (DG), anteromedial nucleus (AM), third ventricle (3V), substantia nigra pars reticularis (SNR). Scale bar: 1mm. **(F)** Body weight tracked from birth, postnatal day 1 (P1).

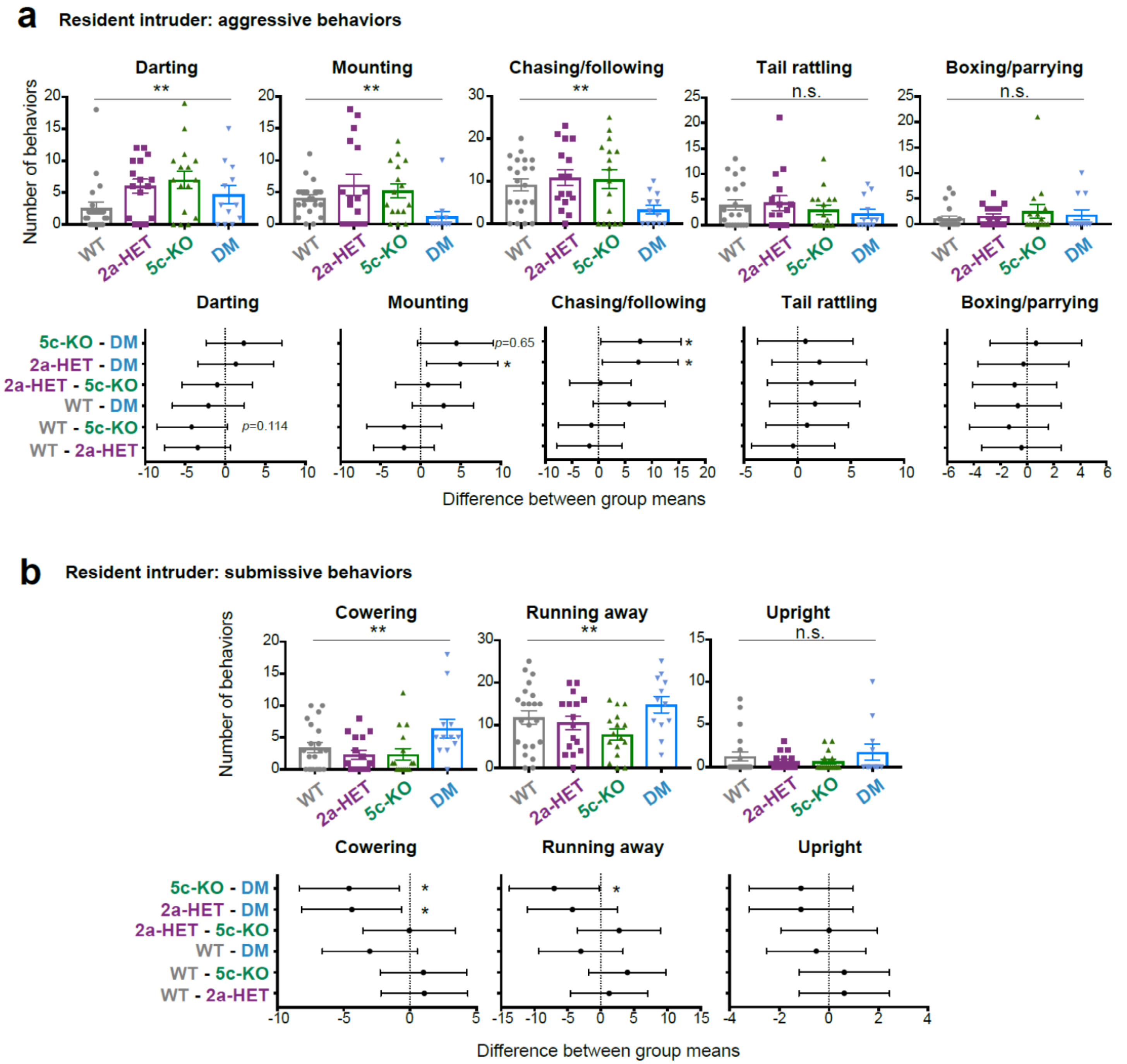

Supplementary Figure 3. Individual behavior types in the resident intruder test. (A) Individual aggressive behaviors (mean ± SEM, ***p* < 0.01 in one-way ANOVA). N.S. depicts no statistical difference. Left panel: average number of all submissive behaviors (mean ± SEM, **p* < 0.05 in one-way ANOVA). Right panel: Difference between group means of submissive behaviors (mean ± 95% confidence intervals, **p*<0.05, ***p*<0.01). **(B)** Individual submissive behaviors (mean ± SEM, ***p* < 0.01 in one-way ANOVA). N.S. depicts no statistical difference. N=21 WT, N=16 *Kmt2a*-HET, N=16 *Kdm5c*-KO, and N=12 DM animals were used for all studies. Differences between group means all aggressive **(A)** and submissive **(B)** behaviors (mean ± 95% confidence intervals, **p*<0.05).

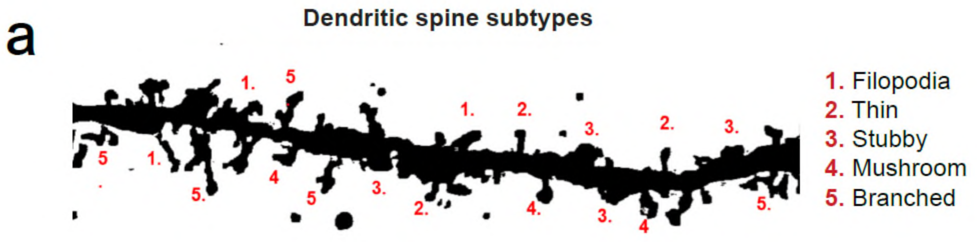

Supplementary Figure 4. Schematic of dendrite spine subtype analysis. (A) Projection image of a dendritic segment from a series of Z stack images derived from BLA pyramidal cells. Numerical marks adjacent to corresponding spine subtypes, represented as: 1. Filopodia, 2. Thin, 3, Stubby, 4. Mushroom, and 5. Branched.

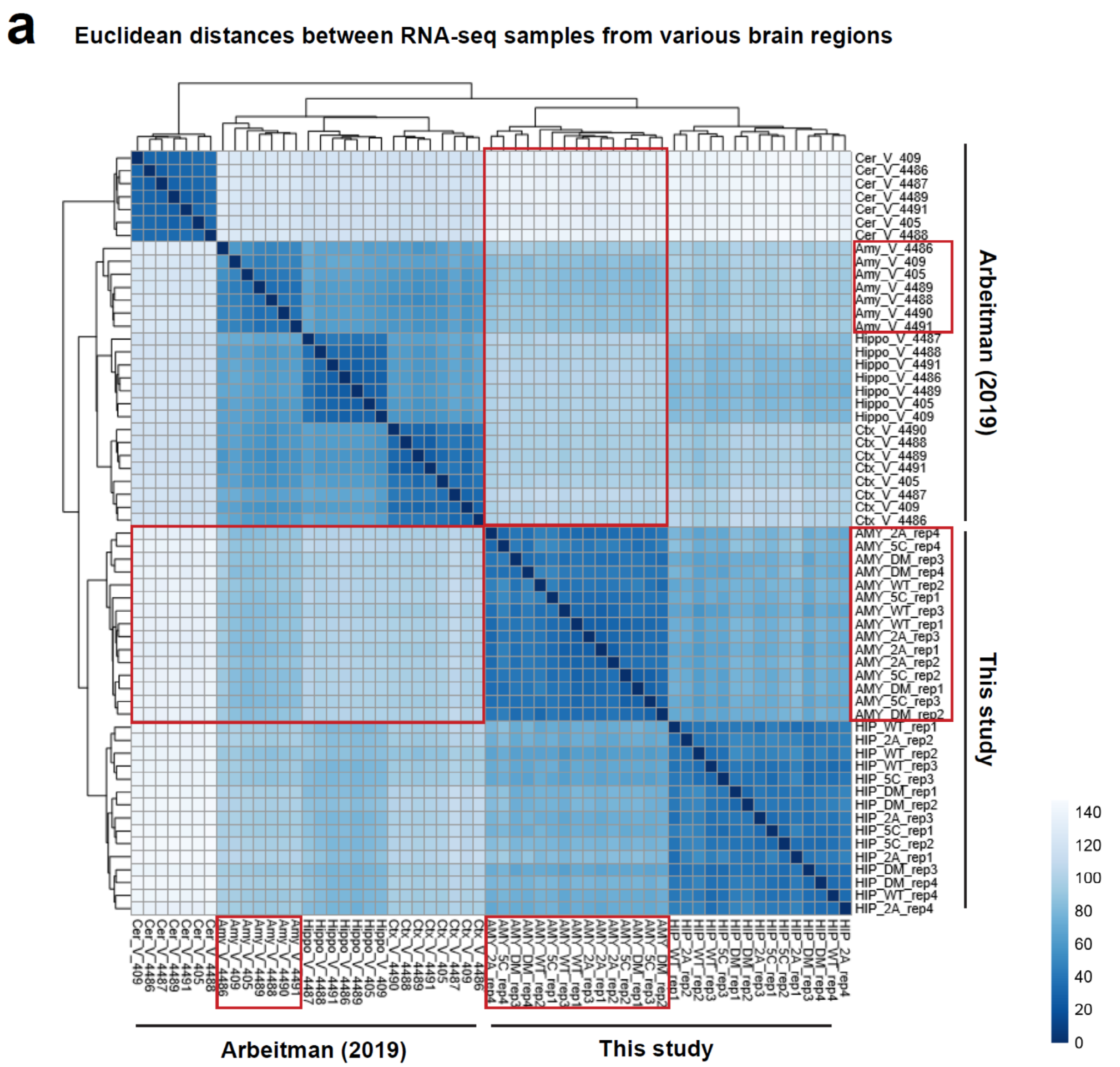

Supplementary Figure 5. Validation of brain microdissection. (A) To validate accuracy of our brain microdissection, we compared our RNAseq data of the hippocampus (HIP) and amygdala (AMY) with those of the Arbeitman paper (5), which involved the hippocampus (Hippo), amygdala (Amy), cerebellum (Cer), cerebral cortex (Ctx). Euclidian distance between all combination of RNA-seq data are plotted (see method). Samples from this study and the Arbeitman study are clustered separately, likely due to difference in experimental procedures and/or sex of mice. Our study used adult male mice, while the Arbeitman study used adult females. Nonetheless, our amygdala samples showed the shortest distance to the Arbeitman amygdala data compared to any other brain tissues (red rectangles). Likewise, our hippocampus data are closest to the Arbeitman hippocampus data among the four brain regions. The data demonstrate that the microdissection of brain regions in the two studies are consistent with each other.

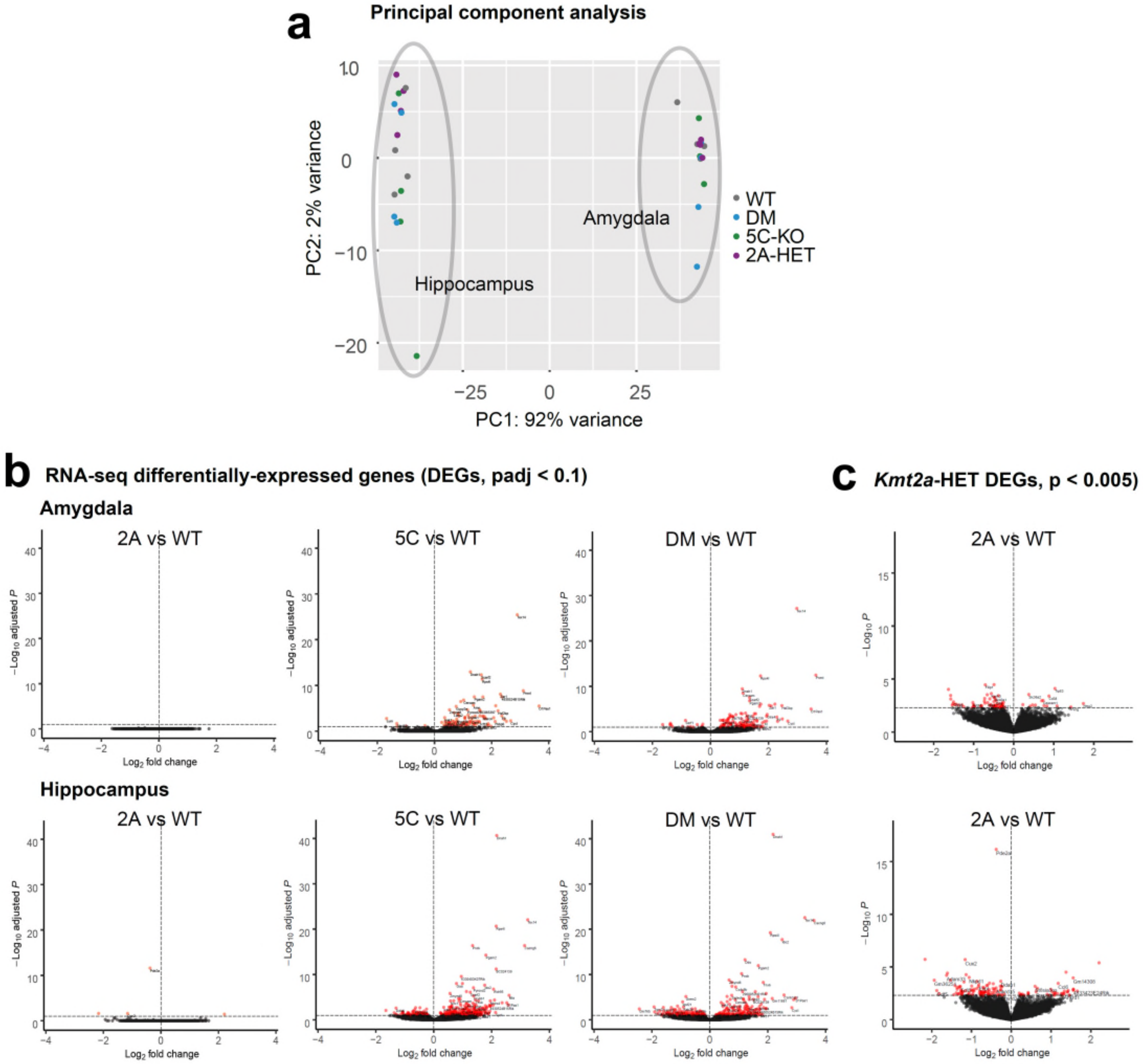

Supplementary Figure 6. Basic analysis of RNA-seq data. (A) PCA analysis of RNA-seq libraries. Tissue types are a stronger segregating factor than genotypes. **(B)** Volcano plot representation of differential expressed genes (DEGs) in mutant vs WT comparisons. Red dots: DEGs with *padj* < 0.1 (see Table S1). **(C)** Mildly dysregulated genes in Kmt2a-HET were recovered with a relaxed threshold.

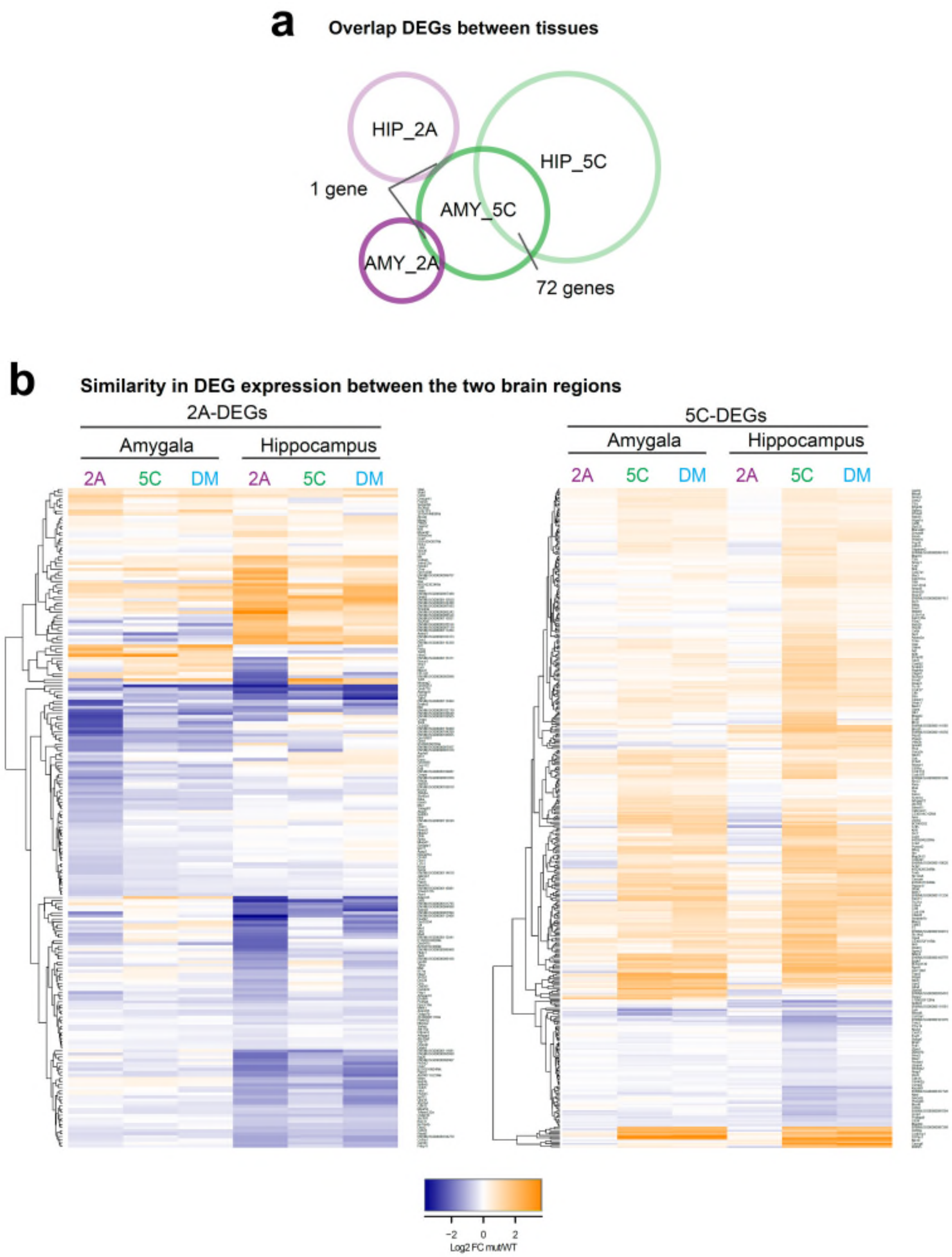

Supplementary Figure 7. Similarity of gene misregulation between the amygdala and hippocampus. (A) Overlap of single mutant DEGs between the amygdala (AMY) and hippocampus (HIP). 5C-DEGs overlap between the two brain regions. **(B)** Heatmap representation of log2 fold changes of 2A-DEGs (left) and 5C-DEGs (right). Overall the patterns of gene misregulation are similar in the two brain regions.

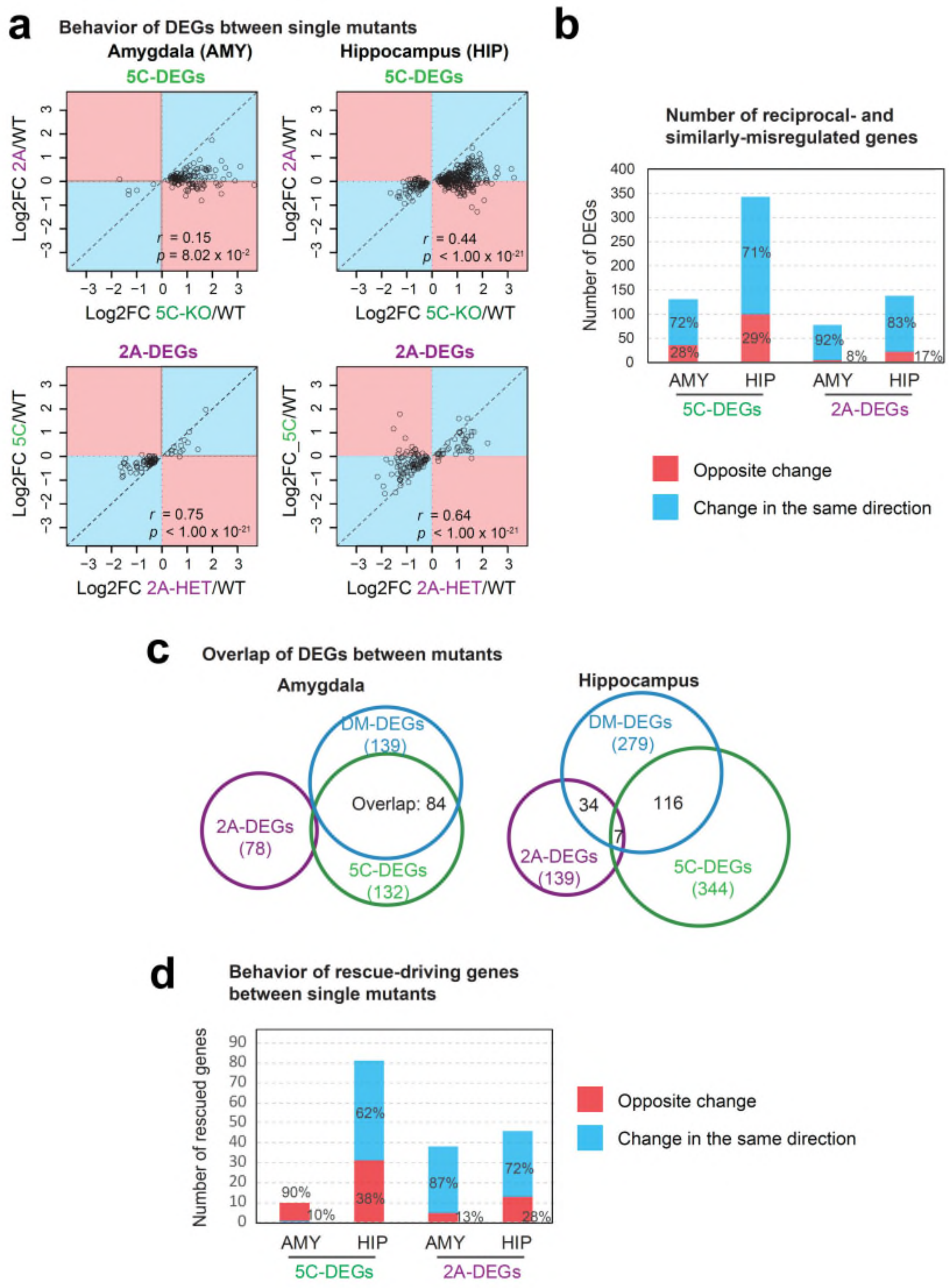

Supplementary Figure 8. Direction of gene misregulation between *Kmt2a*-HET and *Kdm5c*-KO and their overlap in DM brain tissues. (A) Behavior of single-mutant DEGs in the other single mutant. Log2 fold changes of DEGs found in a single mutant were plotted as a function of log2 fold changes in the other single mutant. Red shade covers genes that are regulated in the opposite direction between *Kmt2a*-HET and *Kdm5c*-KO brain tissues. Blue shade covers deregulated genes in the same direction. **(B)** The larger number of genes is dysregulated in the same direction between the two single mutants. **(C)** Overlap of DEGs between mutants. 5C- and DM-DEGs overlap substantially.

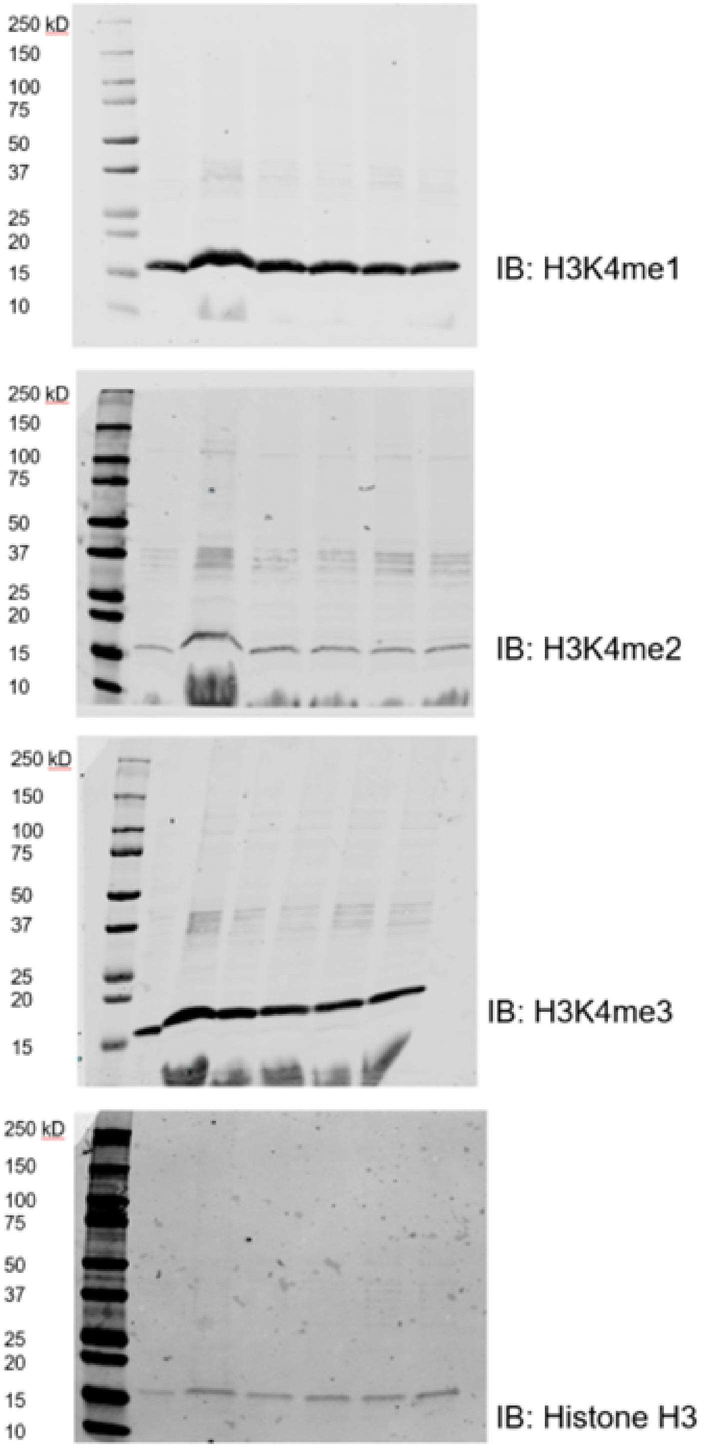

Supplementary Figure 9. Full blots of immunoblot (IB) analyses shown in Figure 6a.

Supplementary Figure 10. Basic characterization of H3K4me3 ChIP-seq data. (A) Validation of H3K4me3 ChIP-seq specificity. Barcode reads originating from spike-in nucleosomes were counted. The two synthetic nucleosomes with the H3K4me3 barcodes dominated all ChIP samples, with H3K4me1/2 nucleosomes rarely detected. **(B)** Volcano plot represents the statistical significance of differentially methylated regions (DMRs). **(C)** MA plots of DMRs revealed signal intensity-dependent misregulation of H3K4me3 in *Kdm5c*-KO, where hypomethylated regions in the mutant were highly methylated in WT.

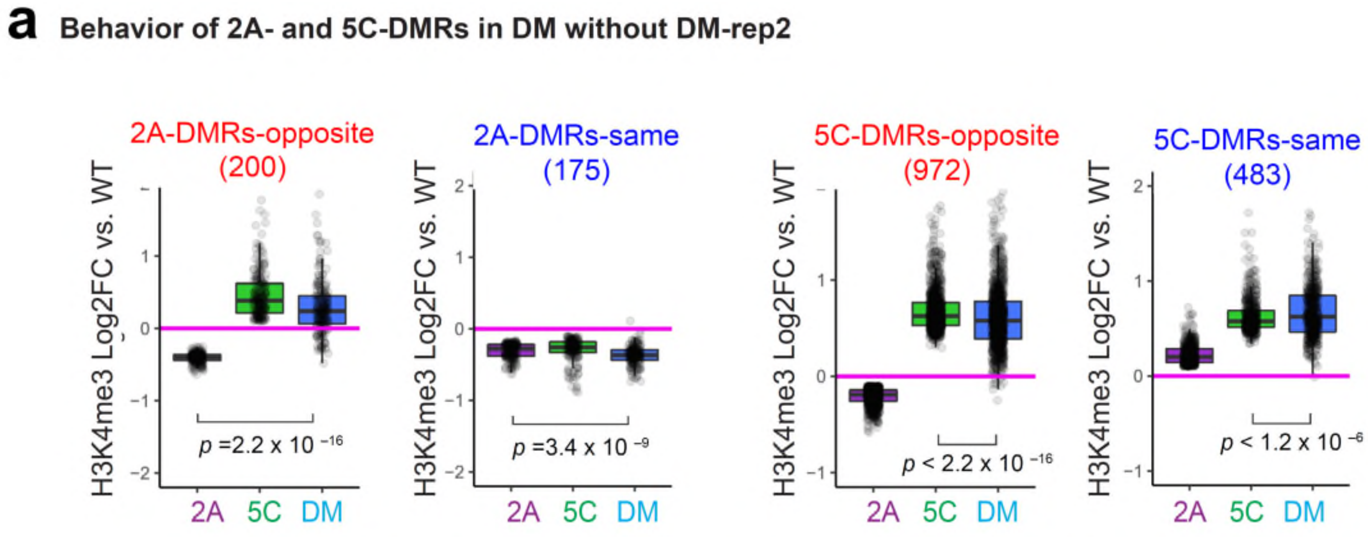

Supplementary Figure 11. Rescue effect without DM rep2. (A) DM rep2 showed strong rescue effect (Figure 6B). To test if H3K4me3 misregulations were alleviated in other DM replicates, we removed DM rep2 and then examined the behavior of single-mutant DMRs in DM. Log2 fold change of DMRs relative to WT were plotted across the three mutants. Boxplot features: box, interquartile range (IQR); bold line, median; gray dots, individual genes. Associated *p* values result from Wilcoxon signed-rank tests. Rescue effects were still evident in DM and also dependent on the direction of misregulation between the single mutants.

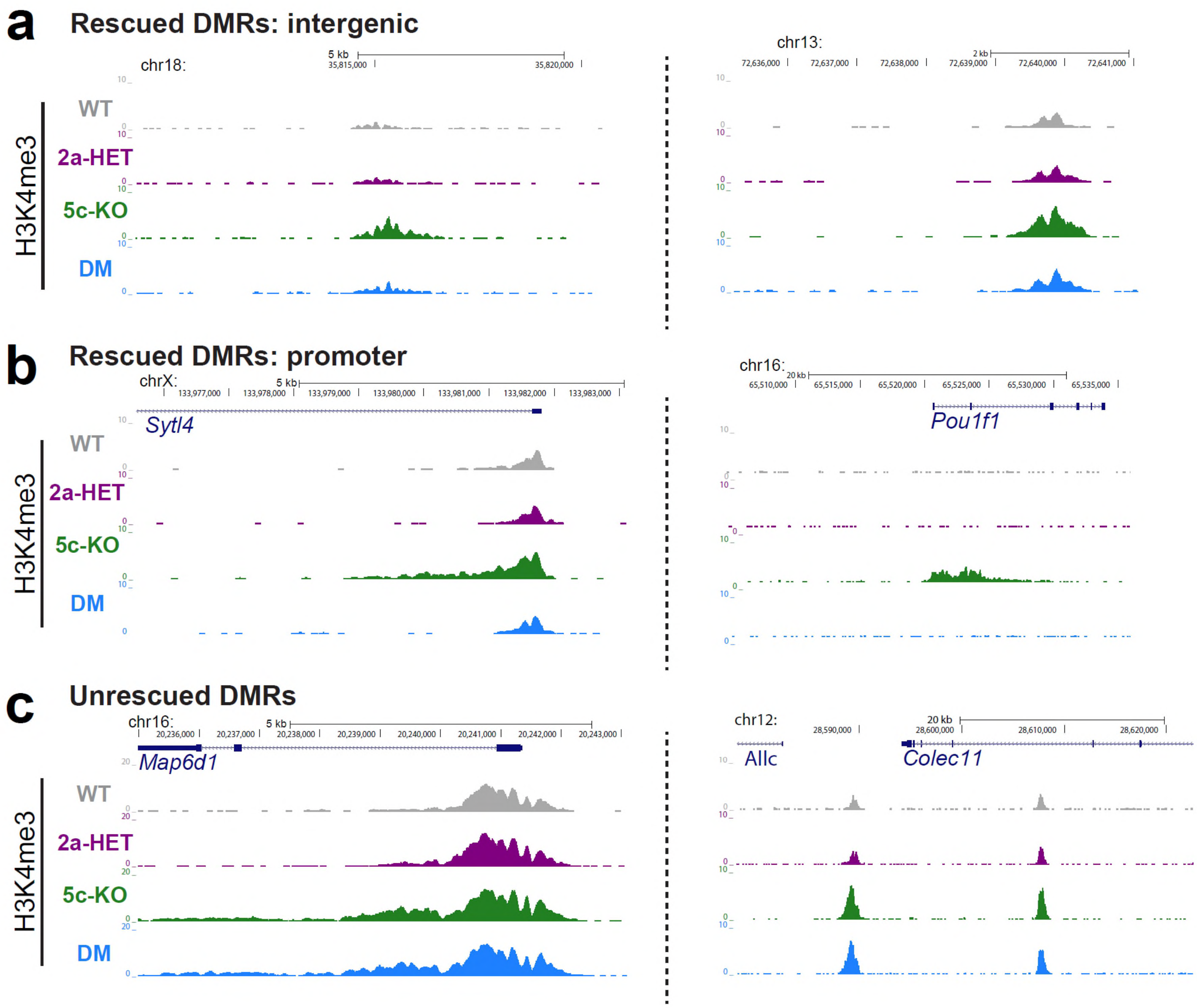

Supplementary Figure 12. Representative loci found in the H3K4me3 ChIP-seq analysis. (A) Representative genome browser view of two representative loci for each of the major genome areas: rescued intergenic DMRs **(A)**, rescued promoter DMRs **(B)**, un-rescued DMRs **(C)**. Represented H3K4me3 patterns are averaged signals of replicates that are normalized to read depth and spike-in nucleosome signals.

References

1. Tasic B, Menon V, Nguyen TN, Kim TK, Jarsky T, Yao Z, et al. Adult mouse cortical cell taxonomy revealed by single cell transcriptomics. *Nat Neurosci.* 2016;19(2):335-46.

2. Lein ES, Hawrylycz MJ, Ao N, Ayres M, Bensinger A, Bernard A, et al. Genome-wide atlas of gene expression in the adult mouse brain. *Nature.* 2007;445(7124):168-76.

3. Kang HJ, Kawasawa YI, Cheng F, Zhu Y, Xu X, Li M, et al. Spatio-temporal transcriptome of the human brain. *Nature.* 2011;478(7370):483-9.

4. Pletikos M, Sousa AM, Sedmak G, Meyer KA, Zhu Y, Cheng F, et al. Temporal specification and bilaterality of human neocortical topographic gene expression. *Neuron.* 2014;81(2):321-32.

5. Arbeitman MN. Maternal Experience Leads to Lasting Gene Expression Changes in Some Regions of the Mouse Brain. *G3 (Bethesda).* 2019;9(8):2623-8.
