# Supplementary material for: Mutually suppressive roles of KMT2A and KDM5C in behaviour, neuronal structure, and histone H3K4 methylation
Source: Commun Biol. 2020 Jun 1;3:278. doi: 10.1038/s42003-020-1001-6 (PMC7264178; doi:10.1038/s42003-020-1001-6)
Supplement: Supplementary file 8 — Peer Review File [file 42003_2020_1001_MOESM8_ESM.pdf]

Editorial Note:*This manuscript has been previously reviewed at another Nature Research journal. The manuscript was considered suitable for publication without further review at Communications Biology*
